# Global services and support for children with developmental delays and disabilities: Bridging research and policy gaps

**DOI:** 10.1371/journal.pmed.1002393

**Published:** 2017-09-18

**Authors:** Pamela Y. Collins, Beverly Pringle, Charlee Alexander, Gary L. Darmstadt, Jody Heymann, Gillian Huebner, Vesna Kutlesic, Cheryl Polk, Lorraine Sherr, Andy Shih, Dragana Sretenov, Mariana Zindel

**Affiliations:** 1 Office for Research on Disparities & Global Mental Health, US National Institute of Mental Health, Bethesda, Maryland, United States of America; 2 National Academies of Sciences, Engineering, and Medicine, Washington DC, United States of America; 3 Department of Pediatrics, Stanford University School of Medicine, Stanford, California, United States of America; 4 UCLA Fielding School of Public Health, WORLD Policy Analysis Center, Los Angeles, California, United States of America; 5 Maestral International, Washington DC, United States of America; 6 Office of Global Health, *Eunice Kennedy Shriver* National Institute of Child Health and Human Development, Bethesda, Maryland, United States of America; 7 HighScope Educational Research Foundation, Ypsilanti, Michigan, United States of America; 8 Research Department of Infection and Population Health, University College London, London, United Kingdom; 9 Autism Speaks, New York, New York, United States of America; 10 Early Childhood Program, Open Society Foundations, London, United Kingdom

## Abstract

Pamela Collins and colleagues explain the research and policy approaches needed globally to ensure children with developmental delays and disabilities are fully included in health and education services.

**Summary points**

- The United Nations Sustainable Development Goals and the UN Convention on the Rights of the Child (CRC) envision an inclusive society in which health and education contribute to the well-being of all. To achieve this vision, children with developmental delays and behavioral, cognitive, mental, and neurological disabilities need greater access to health care, early childhood care and development services, and education.
- Improved population-level detection, alongside screening, assessment, and linkage to evidence-based, intersectoral services in the first years of life, can help maximize capabilities and increase the chances of social inclusion for children with developmental delays and disabilities.
- Educational programs for children with delays and disabilities whose service delivery structure supports the ability of parents to work should be encouraged so that parents can participate in achieving children’s educational goals while also meeting their financial needs.
- Parents and caregivers who receive training in psychosocial interventions and ongoing support can help children with delays and disabilities thrive in family contexts.
- Family mental health influences the developmental trajectory of children. Ensuring that parents and caregivers have access to affordable, quality mental health services helps to prevent poor outcomes for children.
- Rigorous evaluation, continuous quality improvement, and regular monitoring of the programmatic outcomes of services and policy approaches targeting children and caregivers would inform their implementation and serve to disseminate lessons learned from successful policy and program implementation.

## Background

The UN Sustainable Development Goals (SDGs) were formulated based on the principle that people everywhere deserve “equitable and universal access to quality education at all levels, to health care and social protection, where physical, mental and social well-being are assured” [1]. This vision for inclusive healthy societies includes children with developmental delays and cognitive, mental, and neurological disabilities (henceforth developmental delays and disabilities). The UN Convention on the Rights of the Child (CRC) further stipulates that children with disabilities cannot be excluded from free and compulsory primary and secondary education based on their disability [2]. Yet, children with disabilities are more likely to grow up in poverty and to receive less healthcare, early childhood care and development services, and education [3,4]. Caregivers and parents play a central role in facilitating children’s access to early childhood development interventions, including healthcare and education, but must be adequately supported.

Recent analyses highlight the importance of early child development and delineate the conditions that place children at risk for not achieving their developmental potential as well as the interventions and research needed to mitigate this [5–9]. With optimal implementation of existing prevention and care interventions, a subset of children will nevertheless be identified with developmental delays and disabilities of varying severity. Ideally, their caregivers, parents, community structures, and societies can be equipped to accommodate their needs to achieve maximum social inclusion and functioning. This paper identifies research and policy activities that, if implemented, could improve the identification of children with delays and disabilities and the ability of caregivers to help meet their developmental, health, and educational needs. We describe opportunities for research or policy shifts in 5 main areas: identifying children with delays and disabilities, ensuring access to early childhood programs and school programs for children, training and support of parents/caregivers to strengthen their ability to care for their children, supporting caregivers’ ability to work, and ensuring that the mental health needs of caregivers are met.

## Identify children with delays and disabilities

The most recent Global Burden of Disease data estimate that in 2015, there were 3.6 million children aged 1–9 years living with autism and more than 15 million living with idiopathic developmental intellectual disability [10]. These are only 2 of many cognitive, emotional, mental, and neurological disabilities. Yet, neither incidence nor prevalence for the full range of childhood delays and disabilities is well established in global data. Rates of cognitive disabilities linked to infections (e.g., pneumonia, meningitis, encephalitis, and HIV), prematurity and stunting, neonatal encephalopathy, hyperbilirubinemia, prenatal iodine and other nutritional deficiencies, and neural tube defects linked to inadequate folic acid are likely higher in low- and middle-income countries (LMICs) than in high-income countries (HICs) given the numbers of children living in poverty and the distribution of these risk factors [11–14]. The accumulation of adversities, beginning before conception and continuing throughout prenatal and early life, can disrupt brain development, attachment, and early learning [5]. Developmental delays become evident in the first year, worsen during early childhood, and continue throughout life [6, 15].

Over the past decade, population-based studies have measured the prevalence of disabilities across several countries. Utilizing a disabilities module within the 2005–2007 Multiple Indicator Cluster Survey (MICS) [16], 1 study found that 20% of children across 16 LMICs screened positive for at least 1 impairment (range 3% to 45%), and 5%, 12.7%, 2.9%, and 6.2% of children screened positive for a cognitive, language, sensory, or motor impairment, respectively [17]. A more recent estimate derived from predictive modelling in 35 LMICs showed that 81 million 3-to-4-year-olds (33% prevalence) had low cognitive or socioemotional development in 2010 [18]. The proportion of under-5 children in LMICs at risk of not attaining their developmental potential because of extreme poverty and stunting remains high at 43% [5].

Accurate identification of a child’s impairment in the first years of life makes reversal or mitigation of adverse effects more likely [19]. Routine screening can be implemented in primary care with high fidelity, low cost, and acceptable levels of burden [20–23]. Provider training increases screening and identification of developmental delays [24, 25]. Proactive case finding using community informants is also a promising approach [26]. When linked with diagnostic assessment and evidence-based interventions, early detection helps to increase the proportion of children who achieve their developmental potential, fulfill their ability to work and contribute [27], are not raised in institutions, and do not need expensive services later in life [28–30]. Ethical care requires that screening be linked to intervention.

## Increase access to early childhood programs, schooling, and after-school and out-of-school programs

The benefits of early intervention for children with developmental delays and disabilities, families, and communities have been well documented in HICs [28, 31, 32]. A recent review of studies from LMICs provides evidence of similar positive outcomes with early interventions for at-risk children, although research that examines outcomes for children with established disabilities is limited [33]. Scarce human resources for mental, neurological, or developmental pediatric care can limit access to services in LMICs. Task-sharing approaches that provide abbreviated training to less specialized providers for the delivery of evidence-based screening, care, and support interventions can help bridge the resource gap. Researchers in Pakistan screened a large rural community by distributing written descriptions of developmental disorders that included motivational messages and by administering the Ten Questions Screen for disability using an interactive voice response system [34]. Children who screened positive were eligible to work with a network of families equipped with “family champion volunteers” trained in evidence-based interventions outlined in the WHO Mental Health Gap Action Program’s (mhGAP) intervention guide. Significant results included reduced WHO Disability Assessment Schedule global disability scores, lower parent-reported socioemotional difficulties in children, and no diminution of caregiver well-being. Equally important, the family volunteers engaged in more advocacy for children’s education, healthcare, and community inclusion. In another study, nonspecialist health workers in India and Pakistan were trained to coach parents of children with autism to apply strategies for improving parent–child interactions, with an emphasis on communication [35]. Parents and children showed more synchronous communication, and children initiated more communication with the parent.

Access to education remains a critical intervention for children with delays and disabilities, but disparities in educational opportunity, quality, and outcomes persist [36]. Poorer outcomes are related in part to nonenrolment in school, exclusion from participation in classroom activities, and greater likelihood of school dropout [36]. Yet, children with developmental delays and disabilities and their peers without these conditions benefit when early childhood, school, and out-of-school programs are fully inclusive. Education helps break cycles of poverty, potentially for the child with the delay or disability and for siblings who begin a “caregiver career” rather than attending school [3, 37]. The availability of educational programs year-round during the workday plays a key role in ensuring that children and youth with delays and disabilities have the fullest developmental and educational opportunities in settings far better than institutional care can provide. Such programs increase the likelihood that their parents are able to work, support their families, and lead full lives [3]. Integrated education also serves to educate peers on the needs of children with disabilities and provide pathways for interaction and understanding. Table 1 outlines population- and community-level interventions alongside healthcare interventions that can benefit children with delays and disabilities [38].

**Table 1:** Platforms for interventions for children with developmental delays and disabilities.

			Healthcare platforms	
Target areas	Population platform	Community platform	Primary healthcare	First-level hospital care
Children with behavioral, cognitive, emotional, and neurological developmental delays and disabilities	Awareness campaigns to increase mental health literacy and address stigma and discrimination	Training of gatekeepers (frontline workers, police, and teachers) in the early identification of priority disorders	Screening for developmental disorders in children	Diagnosis of childhood mental disorders such as autism and attention-deficit/hyperactivity disorder (ADHD)
	Legislation on the protection of human rights of persons affected by developmental delays and disabilities	Provision of low-intensity psychosocial support for caregivers and referral pathways	Caregiver mental health interventions	Medication for severe symptoms and behaviors
	Child protection laws	Parenting programs in infancy to promote early childhood development	Parent skills training for mental health and developmental disorders	Newborn screening for modifiable risk factors for intellectual disability
		Life skills training in schools to build social and emotional competencies	Psychological treatment, including cognitive-behavioral and family interventions for mood, anxiety, ADHD, and disruptive behavior disorders	Management of severe caregiver depression
		Parenting programs in early and middle childhood (ages 2–14 years)	Improve the quality of antenatal and perinatal care to reduce risk factors associated with intellectual disability	
		Early child enrichment and preschool education programs	Behavior programs including applied behavior analysis and family interventions to address developmental delays and disorders	
		Identification of children with mental, neurological, and substance use disorders in schools		
		Inclusive primary education		
		Individualized education plans		

Reproduced with modifications from *Disease Control Priorities*, Third Edition, Volume 4 [38].

## Train and support parents

Children with delays and disabilities can thrive in family contexts, particularly if parents and caregivers receive proper training and ongoing support. Directive parenting, combined with “sensitive, responsive, and reciprocal outcomes” and a stimulating home and community environment, led to favorable developmental outcomes for infants and children with Down syndrome in 1 study [39]. Conversely, a lack of knowledge about their child’s condition and needs, negative feelings, and lack of support adversely affected parent–child interactions, child behavior, and development. If provided with nurturing and supportive family care, children with delays and disabilities have a better chance of leading healthy and full lives, particularly when such care is provided from early in life. Nurturing care has recently been defined as a stable environment that is sensitive to children’s health and nutritional needs, with protection from threats, opportunities for early learning, and interactions that are responsive, emotionally supportive, and developmentally stimulating [40]. As an overarching concept, nurturing care is supported by an ecosystem of social contexts—from home to parental work, child care, schooling, the wider community, and policy influences [41].

Unfortunately, in many cases parents do not have access to specialized training and programs and/or flexibility in their work environment to care for their developmentally delayed or disabled child. In many countries, this results in high numbers of these children being institutionalized at an early age [42]. In Central and Eastern Europe and the Commonwealth of Independent States (CEE/CIS), a child with any type of disability is nearly 17 times more likely to be institutionalized than a child who does not have a disability [43]. When systemic measures to support children with disabilities and their families are encouraged and developed, institutionalization can often be prevented or reversed. With help from the civil sector, from 2009 to 2012, the Government of Moldova closed 18 institutions and reduced by 62% the number of children living in residential care [44]. Efforts like this http://www.openingdoors.eu/wp-content/uploads/2013/05/Facts-and-figures-Moldova-2015.pdf begin with and are sustained by empowering families and caregivers through making existing support programs more inclusive, developing specialized training programs on caring for atypically developing children, and ensuring that all parents of children with disabilities have access to this critical training. The need for services and support to parents to provide nurturing care and the need for training of health workers and nonspecialists have been identified as research priorities [7].

## Support the ability of parents to work

Worldwide, families caring for children with disabilities have lower incomes because of constraints on employment [45]. The income needs of families with children with developmental delays and disabilities are on average higher than those of families whose children do not have these conditions because of the costs of services and care [46], which are rarely fully covered by public funds. Studies from LMICs and HICs demonstrate that parental attention to children’s health and involvement in education leads to better outcomes for children [45]. To do this while sustaining financial stability requires access to paid leave; yet, globally, marked disparities in access to paid leave for both parents persist [45]. Parents and caregivers employed in informal work sectors likely have even fewer protections. In the absence of adequate leave, wage loss can be significant [47].

Families benefit if there are quality, affordable developmental and educational programs that are accessible year-round while parents work. Moreover, from a societal perspective, the full inclusion of parents of children with special needs in the workforce contributes markedly to broad social inclusion just as fully integrated classrooms for students do. Monitoring these policies at a country level would provide important groundwork for achieving progress (see Table 2). Families may also need additional support, and the evolving data on cash transfers and psychosocial support are an encouraging pathway [40].

**Table 2:** Policies to support children with developmental delays and disabilities and their caregivers.

Policies to ensure:	Policies framed in terms of:	Monitoring and evaluation to determine:
Child access to early diagnosis, support, and care via educational, healthcare, and social welfare systems Inclusive services for children in early childhood care, all grades/levels of education, and after-school and summer programs Education system accommodations, including teacher training, classroom supports, and optimal teacher/aide-to-child ratios Caregiver supports, including caregiver training and paid leave from work to meet child health and education needs Family financial supports, including health insurance coverage and income support/family benefit for the additional cost of caring for a child with a disability Reasonable environmental and other accommodations at school and work Protection from discrimination at school and work	Equity and human rights Developmental appropriateness Inclusive, intersectoral, and mainstream services, supports, and accommodations State-of-the art scientific evidence Needs and rights of all stakeholders, including children, caregivers, providers, and communities	Whether policies grant adequate legal rights to children and families If policies and programs are adequately funded Whether adequate policy enforcement mechanisms exist and are appropriately used How fully and successfully policies are being implemented The extent to which key policy outcomes (e.g., inclusion, protection, accommodation, and evidence-based services) are being achieved

## Treat caregivers with mental health problems

In addition to psychosocial support, the mental health needs of caregivers must be met for them to nurture healthy developmental trajectories in their children. Disabling mental disorders like major depression are prevalent worldwide. The reported prevalence of maternal depression is higher in LMICs (15%–20%) compared to HICs (6%–13%), possibly because of the distribution of social risk factors for maternal depression and the limited healthcare infrastructure and resources for care [48, 49]. Depression limits a mother’s responsiveness to her infant and is associated with inconsistent behavior and less emotional sensitivity to the child [50]. Maternal depression may also lead to early cessation of breastfeeding and undernutrition in the first year of life, lower rates of immunization, higher rates of underweight and stunting [51], and higher rates of childhood illnesses like diarrhea [52]. As compared to children with healthy mothers, infants born to depressed mothers are at a higher risk of poorer long-term cognitive development and delayed motor development; have higher rates of antisocial behavior, hyperactivity, and attention difficulties; and have more frequent emotional problems [48].

There is a growing body of literature indicating that paternal mood also affects child development, and comprehensive provision needs to focus on both parents [53, 54]. Paternal depression is linked to an 8-fold increased likelihood of adverse child–child interactions, with the highest risk of problems with peers among children aged 4–6 years, possibly stemming from negative interactions of depressed fathers with their children [55]. Moreover, parenting children with developmental delays or disabilities can elevate caregivers’ stress, negatively impact quality of life [56, 57], and thus exacerbate the bidirectional adverse effects on both caregiver and child.

Several studies have shown the effectiveness of psychological therapies such as cognitive behavioral therapy and interpersonal therapy in successfully treating and decreasing the symptoms of depression in adults in HICs and LMICs. Where mental health providers are scarce, task shifting is a promising solution to this human resource problem. A meta-analysis of 13 studies that used task shifting to provide psychological therapies aimed at improving parental depression and child health outcomes showed associations between maternal mood and infant health and development as well as positive bidirectional effects of interventions [48, 58]. Across several studies, psychotherapy-based treatments for depressed mothers generally led to improved mother–child bonding, as well as improved language acquisition and fewer externalizing behavior problems in children, but data here are limited [48].

## Conclusion

Managing the needs of children with developmental delays and disabilities and meeting their caregivers’ needs require collaboration across the health system as well as intersectoral cooperation (Table 1 [38]). Ideally, detection and screening would occur at all levels. Referrals for care typically involve educational and behavioral health specialists in HICs, but a small and growing evidence base from LMICs shows that families and nonspecialist providers can also be engaged. Crucially, medical providers must be sensitized to the needs of these children to ensure that they receive adequate preventive and curative healthcare alongside behavioral, social, and educational interventions. Care managers (employed in chronic care models) who can support families and facilitate communication among schools, social services, and healthcare personnel would prove valuable for coordinating care and support.

Whether researchers address questions related to the extent of need or the efficacy of programmatic and policy approaches, they must also keep their focus on equity to achieve the SDGs. This requires careful assessment of whether all groups of children and caregivers are being equally well served. When researchers examine policies, programs, and services, it will be essential to map the extent to which different approaches to promoting equal participation and opportunities for children with disabilities and their families are being implemented and are closing equity gaps (Table 3).

**Table 3:** Research gaps for the identification and care of children with developmental delays and disabilities.

Goal	Research priority areas
Identify children with developmental delays and disabilities and their needs	Develop robust screening and assessment tools to identify children with developmental delays and disabilities across cultural contexts. Develop and evaluate programs to train and supervise providers in screening children and referring them for full assessment and services. Accurately measure the magnitude of the needs of families that have a child with a developmental delay or disability.
Increase access to evidence-based services	Identify successful implementation models for linking children with developmental delays and disabilities to evidence-based services, both within and outside of educational and health systems. Systematically evaluate the feasibility, outcomes, and cost-benefit ratio of early interventions. Assess the feasibility, acceptability, and outcomes of affordable developmental and educational programs that are accessible year-round while parents work. Assess, monitor, and reduce disparities in access to mainstream and specialty services.
Train and support caregivers	Develop and evaluate strategies for training caregivers to care for their developmentally delayed or disabled child. Develop and evaluate structural supports (e.g., flexibility in the work environment and cash transfer programs) to facilitate family care for atypically developing children. Evaluate the implementation and outcomes of mental health screening and care for caregivers of children with a developmental delay or disability. Gain deeper knowledge of how parental mental health influences children’s developmental trajectory and target interventions accordingly. Monitor the effects of new systemic measures to support the families of children with disabilities on outcomes such as preventing institutionalization.
Improve programs and policies	Evaluate programmatic and policy approaches targeting children with developmental delays and disabilities; regularly monitor the extent to which they are in place and achieve the desired results. Analyze the extent to which programs and policies serving broader populations of children and families are equally accessible and beneficial to children with delays and disabilities and their families. Integrate continuous quality improvement approaches into new innovations and initiatives. Evaluate what can be done to improve program and policy implementation, as well as what lessons can be learned from implementation successes.

Progress will require regular monitoring and accountability to ensure that leaders who improve their approaches are rewarded, that countries and localities that lag are supported to improve, that toolkits growing out of the most effective solutions are readily available to all countries, and that approaches for accountability are widely disseminated to the public.

## Abbreviations

ADHD: attention-deficit/hyperactivity disorder
CEE/CIS: Central and Eastern Europe and the Commonwealth of Independent States
CRC: Convention on the Rights of the Child
HIC: high-income country
LMIC: low- and middle-income country
mhGAP: Mental Health Gap Action Program
MICS: Multiple Indicator Cluster Survey
SDG: Sustainable Development Goal
